# Standard gastroscope-guided endotracheal intubation in the lateral position for life-threatening variceal bleeding: a report of technical feasibility and airway safety

**DOI:** 10.1186/s12871-026-03871-3

**Published:** 2026-04-29

**Authors:** Xia Zhang, Chuanyu Sun

**Affiliations:** 1 Department of Anesthesiology, The First Affiliated Hospital of Shandong First Medical University & Shandong Provincial Qianfoshan Hospital, Jinan, China; 2 Shandong Institute of Anesthesia and Respiratory Critical Medicine, Jinan, China; 3 Shandong Provincial Clinical Research Center for Anesthesiology, No. 16766 Jingshi Road, Jinan, Shandong 250014 The People’s Republic of China

**Keywords:** Esophageal variceal bleeding, Lateral position, Tracheal intubation, Standard gastroscope-guided, Emergency endoscopy, Airway management

## Abstract

**Background:**

Patients with acute upper gastrointestinal hemorrhage (UGIH) often present with airway contamination due to hematemesis and gastric reflux. Conventional endotracheal intubation (ETI) in the supine position faces challenges such as limited visualization and high aspiration risk. While securing the airway and controlling bleeding are both critical during resuscitation, simultaneous achievement of these goals remains technically difficult.

**Case presentation:**

A 56-year-old woman with esophageal variceal rupture presented with hemorrhagic shock. After left-lateral positioning and rapid-sequence induction, a cuffed endotracheal tube pre-loaded with a J-tipped guidewire was advanced through the right channel of a bite block into the oropharynx. Under continuous direct visualization provided by a standard diagnostic gastroscope, the tube was steered into the glottis on the first attempt. The scope was then advanced through the tube into the esophagus, permitting immediate endoscopic band ligation of all bleeding varices. The entire process from securing the airway to endoscopic access was completed without apparent delay, with no clinically evident aspiration and with maintained hemodynamic stability.

**Conclusions:**

This case demonstrates the technical feasibility of standard gastroscope‑guided intubation in the lateral decubitus position for airway management in massive upper gastrointestinal (GI) bleeding with hypovolemic shock. The procedure enabled rapid airway control without repositioning‑induced hemodynamic instability, allowed seamless transition to endoscopic hemostasis, and was not associated with visible aspiration or mucosal injury. Considering the limitations of a single case, the approach may reduce risk and appears feasible in this instance. These findings are descriptive and not generalizable. The technique may be considered a potential alternative in selected high‑risk “bloody airway” scenarios when advanced bronchoscopic equipment is unavailable. Further validation in larger studies is needed.

**Supplementary Information:**

The online version contains supplementary material available at 10.1186/s12871-026-03871-3.

## Background

Acute massive upper gastrointestinal bleeding (AMUGIB) is a life-threatening emergency requiring urgent endoscopic hemostasis [1–3]. For patients with active hemorrhage, gastric blood retention and vomiting reflexes substantially elevate peri-procedural aspiration risk, making rapid airway management a core priority of anesthesia-endoscopy collaborative care.

Conventional endotracheal intubation is limited by obscured glottic visibility, increased aspiration risk, and hemodynamic instability induced by mandatory supine repositioning, which delays life-saving endoscopic intervention [4].

While ultrathin gastroscope-guided intubation has been explored previously, its narrow lumen and insufficient suction capacity preclude simultaneous decontamination and guidance [5].

We describe a novel technique using a conventional gastroscope to facilitate lateral-position intubation, which avoids repositioning-related hemodynamic disturbances, shortens the interval between airway protection and endoscopy, and optimizes the procedural workflow.

## Case presentation

A 56-year-old woman presented with acute variceal hemorrhage, manifesting as massive hematemesis, hemodynamic shock (mean arterial pressure[MAP] < 50 mmHg), and progressive encephalopathy (Glasgow Coma Scale[GCS] score 11). She had a medical history of hepatitis B and cirrhosis, and three years previously, she underwent endoscopic variceal ligation for esophageal variceal rupture with bleeding.Laboratory findings were consistent with acute-on-chronic liver failure (hemoglobin [Hb] 4.4 g/dL, international normalized ratio [INR] 1.74), necessitating emergent airway control prior to endoscopic intervention.

### Pre-anesthesia evaluation

The patient presented with hemorrhagic shock, hypotension, and tachycardia. Significant residual blood was likely retained in the esophagogastric lumen. During intubation, there was a high risk of dislodgement and rupture of esophageal variceal thrombi, potentially causing massive rebleeding, along with substantial aspiration risk.

### Anesthesia protocol

1. Volume Resuscitation: Through large-bore peripheral intravenous access, balanced crystalloids and blood products were rapidly infused (1:1:1 ratio), supplemented with titrated norepinephrine (0.05–0.3 µg/kg/min) to maintain MAP > 65 mmHg.
2. Anti-reflux positioning: The left-lateral tilt position (30° reverse Trendelenburg) was strictly maintained from pre-oxygenation through intubation, combined with apneic high-flow nasal oxygenation (HFNO 15 L/min, fraction of inspired oxygen (FiO₂) 100%) for 3 min.
3. Airway Management: To minimize the risk of aspiration, we adopted standard gastroscope-guided endotracheal intubation in the lateral position combined with an apneic rapid sequence induction and intubation (RSII) technique, all of which were carried out during the induction phase.

### Anesthesia management

#### Pre-anesthesia preparation

**Fig. 1:**
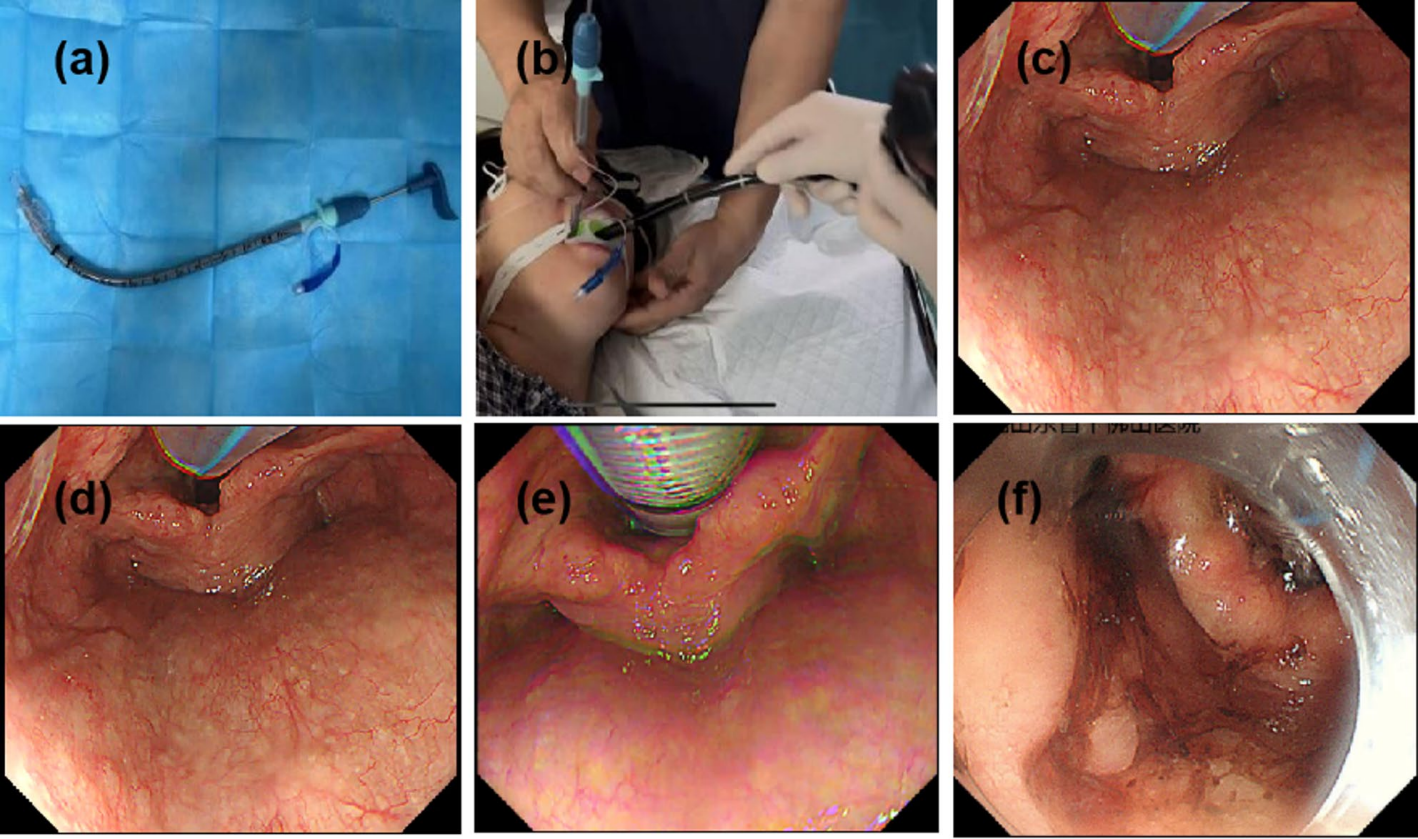
**a** 6.5 mm endotracheal tube preloaded with J-tip guidewire. **b** Dual‑channel workflow: the bite block’s right orifice accommodates the endotracheal tube, while the central orifice allows concurrent gastroscope passage. **c** Endoscopic view of the glottic approach along the mid‑palatal line. **d** Endoscopic visualization of the endotracheal tube tip entering the glottic opening. **e** Oropharynx after tube placement, showing no regurgitation. **f** Endoscopic image showing active esophageal blood reflux into the pharynx

(i) Preload the endotracheal tube with a J-tip guidewire (Fig. 1a).
(ii) Confirm patient cooperation, instruct the patient to open the mouth, and secure the oral airway bite block in position.

#### Anesthesia induction

(i) Administer sequential intravenous etomidate (0.2 mg/kg) and succinylcholine (1 mg/kg).
(ii) Apply the Sellick maneuver (cricoid pressure) immediately upon loss of consciousness.

#### Dual‑channel insertion (Fig. 1b)

(i) After confirming adequate neuromuscular blockade,the anesthesiologist inserts the J‑tip guidewire‑assisted endotracheal tube through the right orifice of the bite block.
(ii) Simultaneously, the endoscopist advances the gastroscope through the central orifice.

#### Endoscopic visualization of the glottis (Fig. 1c)

Advance the gastroscope carefully along the mid‑palatal line, passing the uvula until the anterior aspect of the glottis is visualized.

#### Guided tube advancement (Fig. 1d)

Under direct endoscopic visualization, guide the tube tip into the glottic opening. Withdraw the guidewire upon tracheal entry, inflate the cuff, and connect the tube to the anesthesia circuit.

#### Confirmation of correct placement (Fig. 1e)

Verify correct endotracheal placement by:

(i) Continuous capnography (end‑tidal CO₂ waveform).
(ii) Absence of oropharyngeal regurgitation.

#### Release of Sellick maneuver

Discontinue the Sellick maneuver after confirming secure tube placement (end‑tidal CO₂ >35 mmHg).

#### Observation and management of reflux (Fig. 1f)

Endoscopic visualization reveals active esophageal blood reflux into the pharynx, perform prompt endoscopic suction clear the oropharyngeal field.

### Follow‑up

The patient was discharged home without airway‑related complications,, and at 30‑day follow‑up reported no dysphagia or respiratory symptoms.

## Discussion

This case report describes the application of a standard gastroscope-guided endotracheal intubation technique in the lateral decubitus position for a patient with massive upper GI hemorrhage complicated by severe hypovolemic shock. In this specific scenario, conventional intubation approaches carried a high risk of aspiration due to ongoing regurgitation of blood and gastric contents. The lateral position was selected based on evidence supporting its role in reducing reflux risk [4, 6], and the gastroscope was utilized as a visual guide to secure the airway while simultaneously enabling suction of esophageal blood. This integrated approach facilitated rapid airway control without the need for patient repositioning, which might have exacerbated hypotension.

In this clinical scenario, the primary challenge was the management of a “bloody airway” – a condition where glottic visualization is severely obscured, which significantly increases the difficulty and failure risk of conventional endotracheal intubation.Conventional direct laryngoscopy or video laryngoscopy combined with large-bore suction represents a clinically valid approach, and may achieve comparable intubation speed when performed by experienced practitioners. While video laryngoscopy substantially improves glottic visualization in most airway scenarios, the distal optical lens of the video laryngoscope is highly susceptible to obstruction by blood and secretions within the airway. If visualization is lost during the procedure, the scope must be withdrawn from the airway for decontamination, a process that can directly delay critical time-sensitive emergency intubation.Compared with standard video laryngoscopy, the gastroscope provided direct, real‑time visualization of the pharynx and glottic opening, with the additional capability of continuous suction to clear bloody secretions during tube advancement. In this patient, the entire process from securing the airway to endoscopic access was completed without apparent delay. The technique also eliminated the 2–3 min typically required for supine‑to‑lateral repositioning, thereby minimizing the interval between airway protection and definitive hemostasis. Immediately after intubation, the same gastroscope was advanced into the esophagus to perform endoscopic hemostasis, establishing a continuous workflow that avoided the conventional step of inserting a bite block after laryngoscopic intubation.

We observed that the patient maintained circulatory stability throughout the procedure, with minimal blood pressure and heart rate fluctuations (mean arterial pressure variance < 15%). Furthermore, post‑intubation endoscopy revealed no mucosal injury. These findings are presented as descriptive observations from a single case and should not be interpreted as generalizable conclusions. The absence of tissue traction from laryngoscope‑mediated epiglottis elevation and reduced tracheal stimulation may have contributed to attenuated stress responses, however, this hypothesis requires comparative validation.

This technique should not be viewed as superior to video laryngoscopy, as no direct comparative data exist. Rather, it represents a potential alternative in specific high‑risk scenarios, such as massive GI bleeding with a “bloody airway”, particularly when advanced bronchoscopic equipment is not immediately available. Its reproducibility is supported by the general availability of standard gastroscopes in endoscopic suites. Nevertheless, the technique has a learning curve, and its success depends on collaboration between anesthesiology and gastroenterology teams. Further validation through multicenter clinical trials is warranted, and inclusion in standardized training protocols for endoscopic hemostasis in massive GI hemorrhage is suggested.

## Conclusions

This case report describes a technique for airway management in massive upper GI bleeding with hypovolemic shock: standard gastroscope‑guided endotracheal intubation in the lateral decubitus position. The approach combines the anti‑reflux benefit of lateral positioning with the gastroscope’s real‑time visualization and continuous suction, enabling rapid airway control in a “bloody airway” without repositioning‑induced hemodynamic instability. In this case, the procedure was associated with stable hemodynamics, absence of visible aspiration, and no mucosal injury. The same gastroscope was then used for immediate endoscopic hemostasis, establishing a continuous workflow.

As a single descriptive observation, these findings are not generalizable. This technique is not claimed to be superior to video laryngoscopy but represents a potential alternative in specific high‑risk scenarios, particularly when advanced bronchoscopic equipment is unavailable. Reproducibility depends on the availability of standard gastroscopes and collaboration between anesthesiology and gastroenterology teams. Further multicenter trials are warranted to validate its safety and applicability.

## Supplementary Information

Supplementary Material 1.

Supplementary Material 2.

Supplementary Material 3.

## Data Availability

The datasets are available from the corresponding author on request. All data is provided within the manuscript and supplementary information files.

## Abbreviations

AMUGIB: Acute massive upper gastrointestinal bleeding
GI: Gastrointestinal
UGIH: Acute upper gastrointestinal hemorrhage
ETI: Conventional endotracheal intubation
MAP: Mean arterial pressure
GCS: Glasgow Coma Scale
Hb: Hemoglobin
INR: International normalized ratio
HFNO: High-flow nasal oxygenation
FiO₂: Fraction of inspired oxygen
RSII: Rapid sequence induction and intubation
CO₂: Carbon dioxide
