# Supplementary material for: Standard gastroscope-guided endotracheal intubation in the lateral position for life-threatening variceal bleeding: a report of technical feasibility and airway safety
Source: BMC Anesthesiol. 2026 Apr 29;26:358. doi: 10.1186/s12871-026-03871-3 (PMC13267759; doi:10.1186/s12871-026-03871-3)
Supplement: Supplementary file 1 — Supplementary Material 1. [file 12871_2026_3871_MOESM1_ESM.pdf]

CARE case report guidelines

CARE Checklist of information to include when writing a case report

(cc) BY-NC-ND

Topic	Item	Checklist item description	Reported on Line
Title	1	The diagnosis or intervention of primary focus followed by the words "case report"	Line 1-3
Key Words	2	2 to 5 key words that identify diagnoses or interventions in this case report, including "case report"	Line 43-45
Abstract (no references)	3a	Introduction: What is unique about this case and what does it add to the scientific literature?	Line 31-41
	3b	Main symptoms and/or important clinical findings	Line 20-21
	3c	The main diagnoses, therapeutic interventions, and outcomes	Line 21-29
	3d	Conclusion—What is the main "take-away" lesson(s) from this case?	Line 31-41
Introduction	4	One or two paragraphs summarizing why this case is unique (**may include references**)	Line 58-61
Patient Information	5a	De-identified patient specific information.	Line 62-69
	5b	Primary concerns and symptoms of the patient.	Line 71-75
	5c	Medical, family, and psycho-social history including relevant genetic information	Line 66
	5d	Relevant past interventions with outcomes	Line 66-68
Clinical Findings	6	Describe significant physical examination (PE) and important clinical findings.	Line 71-75
Timeline	7	Historical and current information from this episode of care organized as a timeline	Line 70-87
Diagnostic Assessment	8a	Diagnostic testing (such as PE, laboratory testing, imaging, surveys).	Line 62-69
	8b	Diagnostic challenges (such as access to testing, financial, or cultural)	Line 70-75
	8c	Diagnosis (including other diagnoses considered)	Line 63
	8d	Prognosis (such as staging in oncology) where applicable	Not applicable
Therapeutic Intervention	9a	Types of therapeutic intervention (such as pharmacologic, surgical, preventive, self-care)	Line 85-88
	9b	Administration of therapeutic intervention (such as dosage, strength, duration)	Line 89-120
	9c	Changes in therapeutic intervention (with rationale)	Not applicable
Follow-up and Outcomes	10a	Clinician and patient-assessed outcomes (if available)	Not applicable
	10b	Important follow-up diagnostic and other test results	Line 130-132
	10c	Intervention adherence and tolerability (How was this assessed?)	Line 111-113
	10d	Adverse and unanticipated events	Not applicable
Discussion	11a	A scientific discussion of the strengths AND limitations associated with this case report	Line 133-182
	11b	Discussion of the relevant medical literature **with references**.	Line 136-139
	11c	The scientific rationale for any conclusions (including assessment of possible causes)	Line 154-164
	11d	The primary "take-away" lessons of this case report (without references) in a one paragraph conclusion	Line 183-199
Patient Perspective	12	The patient should share their perspective in one to two paragraphs on the treatment(s) they received	Not applicable
Informed Consent	13	Did the patient give informed consent? Please provide if requested	Yes ☑ No ☐
